# Evaluation of the Biocontrol Potential of Endophytic Fungus *Fusarium solani* CEF559 against *Verticillium dahliae* in Cotton Plant

**DOI:** 10.1155/2019/3187943

**Published:** 2019-12-20

**Authors:** Feng Wei, Yun Zhang, Yongqiang Shi, Hongjie Feng, Lihong Zhao, Zili Feng, Heqin Zhu

**Affiliations:** ^1^State Key Laboratory of Cotton Biology, Institute of Cotton Research of Chinese Academy of Agricultural Sciences, Anyang, Henan 455000, China; ^2^Zhengzhou Research Base, State Key Laboratory of Cotton Biology, Zhengzhou University, Zhengzhou 455001, Henan, China

## Abstract

Verticillium wilt of cotton, caused by the soilborne pathogen *Verticillium dahliae*, is one of the most serious diseases of cotton worldwide. Increased concerns about the side effects of synthetic pesticides have resulted in greater interest in developing biocontrol strategies against Verticillium wilt. We evaluated a *Fusarium solani* CEF559 isolate, obtained from the endosphere of healthy cotton plants, for its biocontrol potential against *V*. *dahliae in vitro* and *in vivo*. In addition to disease assessment, three key genes in the lignin metabolism pathway and four pathogenesis-related (PR) genes were monitored using qRT-PCR. In the laboratory tests, *F. solani* CEF559 inhibited *V*. *dahliae* colony growth by 75% and sporulation by nearly 80% and completely suppressed conidial production. However, volatile metabolites from CEF559 did not affect *V. dahliae* colony growth. In the greenhouse study, CEF559 significantly reduced wilt development, with a control efficacy greater than 60% when assessed 25 days postinoculation. In a field experiment, CEF559 reduced wilt development, with the efficacy ranting from 30.1% to 56.3%. PR genes and those key genes in the lignin metabolism pathway were transiently upregulated in the cotton roots pretreated with CEF559 when subsequently inoculated with *V. dahliae*, compared with those plants inoculated with *V. dahliae* only. Moreover, CEF559 inhibited *V. dahliae* colonisation of both the roots and hypocotyls. The present results suggest that this cotton endophytic fungal strain, *F*. *solani* CEF559, confers protection against *V*. *dahliae*.

## 1. Introduction

*Verticillium dahliae* Kleb. is a soilborne plant pathogenic fungus causing wilt disease on more than 200 plant species [1, 2], including cotton (*Gossypium hirsutum* L.). Yield losses in affected cotton plants were estimated to be around 80% in *V*. *dahliae*-infested soil [1]. The pathogen can survive in the soil as melanized microsclerotia without a host plant for at least 14 years [3]. Incidence of Verticillium wilt of cotton increases with increasing soil inoculum density [4]. Controlling Verticillium wilt in cotton is difficult because of the inaccessibility of *V. dahliae* propagules during the infection by usual control agents, long-term survival of pathogen propagules in the field, and its broad host range [1, 2, 5]. Soil fumigation with pesticides has been an indispensable tool for controlling soilborne pathogens; however, several of these fumigants are already banned under the Montreal Protocol [6]. The remaining fumigants (e.g., chloropicrin and dazomet) are expensive and face an uncertain future due to legislations restricting their use.

Over the last three decades, many nonpesticide strategies have been evaluated and used to control Verticillium wilt, including crop rotation, biofumigation, soil amendment with green manures or organic materials, anaerobic soil disinfestation, and soil solarization [5, 6]. However, all these methods have their own specific limitations [5]. For example, crop rotation with nonhosts of *V*. *dahliae* has not been adopted in the Xinjiang province (the main cotton production region) in China—due to the difficulties in changing cropping systems and saline-alkaline soils. The control efficacy achieved with biofumigation in naturally infested soil was variable and often low because of the low rate of releasing 2-propenyl isothiocyanate [7].

Using biological control agents (BCAs) to manage soilborne diseases is another alternative to chemical control [5]. Microbial-based products have been used commercially in agriculture for over 120 years, but only recently they have received increased attention due to their potential in providing environmentally safe disease control [8]. Biocontrol using antagonists to control Verticillium wilt has been practiced worldwide, such as the use of *Bacillus* spp. [9, 10], *Pseudomonas fluorescens* [9], *Streptomyces plicatus* [11], nonpathogenic *Fusarium oxysporum* [12], *Piriformospora indica* [13], *Trichoderma* spp. [14], *Purpureocillium lilacinum* [15], and *Paenibacillus alvei* [16]. However, examples of successful biocontrol products against soilborne diseases in commercial crop production under open field conditions are limited [17]. Although antagonistic interactions among BCAs are more likely to occur than synergistic interactions, the efficacy of combined use of BCAs is expected to be greater than individual components [18]. Therefore, applying multiple BCAs in the same niche with multiple functional attributes may increase biocontrol efficacies over the use of single biocontrol products.

Plant endophytes inhabit living tissue of plants and do not cause any apparent or detectable symptoms in the host. They include fungi, bacteria, and actinomycetes [19]. Host plants may sometimes benefit from coexisting endophytes that provide resistance to biotic and abiotic stresses [20, 21]. For example, some endophytes are able to inhibit pathogen growth through antibiosis, competition, and parasitism [22] or by induced systemic resistance [23]. We isolated 642 endophytic fungal strains from healthy cotton plants [24] and demonstrated that *Penicillium simplicissimum*, *Leptosphaeria* sp., *Talaromyces flavus*, and *Acremonium* sp. can reduce wilt development in cotton [25].

Nonpathogenic *Fusarium* spp. are gaining interest in agriculture as BCAs to control soilborne plant pathogens [12,26–30]. However, there is little information about using *Fusarium* spp. as a BCA against cotton Verticillium wilt. Thus, we carried out investigations on both *in vitro* and *in vivo* biocontrol potential of a cotton endophyte isolate of *F. solani* (CEF559) against cotton wilt. Furthermore, we studied the expression of pathogenesis-related genes and key genes in the lignin metabolism pathway in cotton against *V. dahliae* following inoculation with *F*. *solani* CEF559.

## 2. Materials and Methods

### 2.1. Fungal Isolates

A *Fusarium solani* strain, CEF559, was an endophyte, isolated from a healthy cotton plant and identified by the rDNA ITS sequences [24] and complementary elongation factor 1-α gene (GenBank accession number: KU382502). A highly aggressive defoliating isolate of *V*. *dahliae* Vd080 isolated from a cotton plant was used in this study [31].

#### 2.1.1. Preparation of Fungal Liquid Culture

Both *V*. *dahliae* Vd080 and *F*. *solani* CEF559 were cultured separately in liquid Czapek-Dox medium for 6 days at 25°C in a shaker at 150 rpm. Conidia were harvested from the liquid medium and resuspended to approximately 1 × 10^7^ spores·mL^−1^ in sterile distilled water for further experimentation.

#### 2.1.2. Preparation of Fungal Solid Culture

An 800 mL Claisen flask containing broken maize-sand medium (vol/vol = 1 : 1) was steam-sterilized at 121 kPa for 45 min and then inoculated with 10 mL spore suspension of *F*. *solani* CEF559. The flask was then incubated at 25°C for 7 days and shaken vigorously by hand for 2 min every day. The dried maize-sand culture was grounded to particles of 1∼2 mm in size and stored at 4°C until further use.

#### 2.1.3. Preparation of Cell-Free Culture Filtrate

*F*. *solani* CEF559 agar-mycelium disks (5 mm diameter), taken from the edge of an actively growing fungal colony, were inoculated to a 500 mL Erlenmeyer flask containing 300 mL liquid Czapek-Dox medium, and the culture was maintained at 25°C on a shaker incubator at 150 rpm for 5 days. The crude culture filtrate was filtered with three layers of filter paper, and the filtrate was filter-sterilized through a 0.2 *μ*m millipore filter [32].

### 2.2. Evaluation of the Antagonistic Activity of *F. solani* CEF559

#### 2.2.1. Dual Culture Assay

Antifungal assay was performed in 9 cm Petri dishes, each containing 20 mL PDA medium. A 5 mm diameter Vd080 mycelial disk was placed at the center of a PDA plate, and four 5 mm diameter mycelial disks of *F. solani* CEF559 were placed next to the Vd080 mycelial disk (south, west, east, and north, 25 mm from the Vd080 mycelial disk). The plate was then immediately sealed with plastic film and incubated in the dark at 25°C for 14 days. *V*. *dahliae* colony diameter was measured in two directions daily. A plate inoculated with *V. dahliae* alone served as the control. There were three replicate plates, and the experiment was repeated twice. The percentage of growth inhibition (IR) 6 days after inoculation was calculated as follows: IR (%) = [(control colony diameter − treatment colony diameter)/control colony diameter] × 100.

#### 2.2.2. Nonvolatile Metabolite Inhibitory Assay

Production of nonvolatile metabolites was estimated by placing a 5 mm CEF559 mycelial disc centrally on two layers of sterilized cellophane covering PDA medium. The plates were incubated for 6 days at 25°C before the two layers of cellophane, CEF559 cultures were removed, and a 5 mm disc of *V. dahliae* Vd080 was placed at the center. The control plates were only inoculated with *V*. *dahliae* Vd080. The dishes were incubated for a further 12 days. The percentage of growth inhibition after inoculated with *V*. *dahliae* Vd080 was calculated as described above. There were three replicate plates, and the experiment was repeated twice.

#### 2.2.3. Volatile Metabolite Inhibitory Bioassay

Inhibition of *V. dahliae* mycelial growth by volatiles of CEF559 was tested using the double-dish method [33]. A mycelial agar plug of CEF559 was placed onto PDA in a Petri dish and incubated at 25°C for 7 days. Another fresh PDA Petri dish containing a mycelial agar plug (5 mm diameter) of *V*. *dahliae* Vd080 was placed inversely over the Petri dish containing the 7-day-old culture of CEF559; this double-dish set was immediately sealed with parafilm. In the control treatment, a PDA Petri dish inoculated with *V*. *dahliae* Vd080 was placed inversely over another Petri dish containing PDA but without CEF559 to make a double-dish set. There were three double-dish sets serving as three replicates. Diameter of the *V*. *dahliae* Vd080 colony in each double-dish set was measured 7 days after incubation at 25°C, and the percentage of growth inhibition was calculated using the method as described above. The experiment was performed three times.

#### 2.2.4. Antifungal Activity of *F*. *solani* CEF559 Filtrate on Sporulation and Spore Germination of *V*. *dahliae*

Cell-free culture filtrate of CEF559 (20 mL) was added to a 50 mL sterile Erlenmeyer flask, and then 100 *μ*L spore suspension (1 × 10^7^ spores·mL^−1^) of Vd080 cultured in liquid Czapek-Dox medium was added. The same volumes of liquid Czapek-Dox medium and spore suspension of Vd080 were added to another flask as the control. The flasks were shaken at 150 rpm for 4 days at room temperature (25°C). Spore concentrations were estimated with a hemocytometer on days 4 and 6. This test was repeated twice.

#### 2.2.5. Antifungal Activity of *F*. *solani* CEF559 Metabolites on Spore Germination of *V. dahliae*

*V*. *dahliae* Vd080 spores were harvested 5 days postinoculation (dpi) in liquid Czapek-Dox medium. Conidia were purified using a previously published method [24]. Spore concentration was adjusted to 2 × 10^3^ spores·mL^−1^; 100 *μ*L of the spore suspension was evenly spread onto a PDA plate. In order to determine the effect of high temperature on the activity of *F*. *solani* CEF559 metabolites, a PDA plate with metabolites of *F*. *solani* CEF559 was autoclaved at 121°C for 20 min and then inoculated with *V*. *dahliae* Vd080. The control plates were only inoculated with *V*. *dahliae* Vd080. Then, plates were maintained at 25°C and germination was assessed under a microscope 3 dpi. Germination inhibition was calculated as follows: GIR = (germination in control − germination in the CEF559 treatment)/germination in control × 100%. There were three replicate plates, and the experiment was repeated twice.

#### 2.2.6. Antifungal Activity of Crude Protein of CEF559 on *V*. *dahliae*

The crude protein was precipitated from cell-free culture broth supernatant with 60% saturated ammonium sulphate (w/v) that was stirred overnight at 4°C to allow protein precipitation. The precipitated proteins were pelleted by centrifugation at 6000 rpm for 30 min at 4°C, dissolved in a 1/10 (v/v) phosphate buffer (0.2 M, pH 7.0), and dialysed for 12 h to remove ammonium sulphate. Some of the crude proteins were dissolved in phosphate buffer (0.2 M, pH 7.0), and their antifungal activity was tested against *V. dahliae*. The PDA plate was spread inoculated with *V. dahliae* Vd080 spore suspension, and then two wells were drilled symmetrically. The phosphate buffer saline (PBS) containing proteins (2 mg·mL^−1^) was added to one well, and the PBS-treated hole as the control. The plates were then incubated at 25°C for 3 days. There were three replicate plates, and the experiment was repeated twice.

### 2.3. Suppressive Effect of CEF559 on *V. dahliae* in Greenhouse

The efficacy of CEF559 spore suppression against *V*. *dahliae* was studied in a greenhouse. Plants were inoculated with CEF559 via either liquid or solid culture inoculation.

#### 2.3.1. Liquid Culture Inoculation

Seeds of cv. Jimian11 (highly susceptible to *V. dahliae*) were surface-disinfected in 1% NaClO solution for 5 min and rinsed three times with sterile distilled water and then dried and sown in paper pots (6 cm in diameter and 10 cm in height, made of newspaper) filled with autoclaved substrate (vermiculite/sand [*V* : *V*] = 3 : 2) [25]. The pots were placed on a plastic tray (35 × 45 cm). Two weeks after sowing, each pot was thinned to leave five plants. The roots of cotton plants were inoculated with *F*. *solani* CEF559 (1 × 10^7^ spores·mL^− 1^, 50 mL/pot) after the first true leaves presented. Four days after inoculation with CEF559, the plants were inoculated with spore suspension of *V. dahliae* strain Vd080 (1 × 10^7^ spores·mL^−1^, 10 mL/pot). The same volume of liquid Czapek-Dox medium instead of CEF559 spore suspension was used in the control treatment.

#### 2.3.2. Solid Culture Inoculation

The solid culture medium of CEF559 (1 × 10^8^ spores·g^−1^) was mixed with the sterile vermiculite and sand (*V* : *V* = 3 : 2) in a ratio of 2 : 98 (*V* : *V*) [25]. Cotton seedlings with one true leaf were inoculated with *V*. *dahliae* Vd080 spore suspension (1 × 10^7^ spores·mL^−1^) following a published method [34].

There were six pots for each treatment, each with 3∼5 seedlings. Seedlings from both the liquid and solid culture inoculation experiments were maintained in a greenhouse under a 12 h/12 h day/night cycle at 25∼28°C. Disease severity of each seedling was recorded 21 days dpi on the following five categories: 0 = healthy, no symptoms on leaves; 1 = one or two cotyledon leaves showing symptoms; 2 = a single true leaf showing symptoms; 3 = more than two leaves showing symptoms; and 4 = plant dead. An overall disease index for each treatment was estimated as follows: disease index =[(0*n*_0_ + 1*n*_1_ + 2*n*_2_ + 3*n*_3_ + 4*n*_4_)/4*n*] × 100, where *n*_0_–*n*_4_ were the numbers of plants with each of the corresponding disease scores, and *n* was the total number of plants assessed for each treatment.

The disease control efficacy was then estimated as follows: efficacy (%) = [disease index_(control)_ − disease index_(treatment)_]/disease index_(control)_ × 100. Each experiment was repeated twice.

### 2.4. Effect of *F. solani* CEF559 on Cotton Emergence and Biomass under Greenhouse Conditions

The effect of *F. solani* CEF559 on cotton emergence and biomass was evaluated in a greenhouse. Paper pots were inoculated with CEF559 solid culture as described above, and the control was mock-inoculated with maize-sand medium. After sterilization for 5 min in a 1% NaClO solution, seeds of cotton cv. Jimian11 were rinsed three times in sterile distilled water and air-dried in a flow cabinet. For each treatment, there were six pots, each with five seedlings. Five days after sowing, the numbers of cotton seedlings in each pot were counted and thereafter every 2 days until no more emergence. Plant height, root length, and fresh weight were measured 20 days after sowing. The assay was repeated twice.

### 2.5. Quantification of *V. dahliae* in Cotton Roots and Hypocotyls

To quantify pathogen colonisation level in roots and hypocotyls following CEF559 treatments, *V*. *dahliae* biomass in cotton plants was estimated by quantitative PCR (qPCR). Ten (cv. Jimian11) seedlings were harvested 21 dpi with *V*. *dahliae* Vd080 for each of the following three treatments of liquid culture inoculation: (i) root inoculated with 50 mL CEF559 cell-free culture and inoculated with 10 mL spore suspensions of Vd080 4 days later; (ii) root inoculated with Vd080 and inoculated with CEF559 4 days later; and (iii) root inoculated with Vd080 alone. DNA was extracted from roots and hypocotyls of all 11 plants using the CTAB method and quantified by NanoDrop 2000. For each sample, 200 ng of DNA was used in the qPCR with the primer pair Vd-F (CCGCCGGTCCATCAGTCTCTCTG-TTTATAC)/Vd-R (CGCCTGCGGGACTCCGATGCGAG-CTGTAAC) [35]. The cotton *ubiquitin* gene was used for normalization with the primer ubiquitin-F/R (Table 1). The experiment was repeated twice.

### 2.6. Control Efficacy of CEF559 against Verticillium Wilt in Field

Field experiments were conducted to evaluate the efficacy of CEF559 against Verticillium wilt. Two fields with a history of severe wilt infestation, located in Anyang (36°05′19.46″N, 114°30′47.21″E) and Akesu (41°10′04.29″N, 80°32′31.92″E), China, were used. Both fields had more than 10 years of continuous cotton crops. There were two treatments: with or without (control) CEF559 inoculation. Solid cultures of CEF559 (1 × 10^8^ spores·g^−1^) were scattered into seed furrows at the rate of 20 g per meter of the furrow; the sterile maize-sand medium was used as control. Surface-sterilized cotton seeds (cv. Jimian11) were sown in the field. A completely randomized design was used with three replications per treatment. Each replicate plot had 6 rows of 8 m in length, containing about 200 cotton plants. Wilt severity of each plant was recorded 60, 80, and 100 days after sowing on a scale of 0 to 4: 0 = no symptoms, 1 = ≤33%, 2 = >33% and ≤66%, 3 = >66% and ≤99%, and 4 = 100% leaves with wilt symptoms. Disease index for each plot was then calculated as described above.

### 2.7. Expression Analysis of Pathogenesis-Related (PR) Genes and Key Genes in the Lignin Metabolism Pathway using qRT-PCR

The relative transcript levels of PR genes and key genes in the lignin metabolism pathway were determined with a quantitative reverse transcription PCR (qRT-PCR) method. Cotton seedlings were first inoculated with CEF559 (1 × 10^7^ spores·mL^−1^, 50 mL per paper pot) at the one true leaf stage and then with *V*. *dahliae* Vd080 (1 × 10^7^ spores·mL^−1^, 10 mL per pot) 4 days later. For each sampling time point (1, 2, 3, 4, and 7 dpi with *V*. *dahliae* Vd080), root samples of each biological replicate and ten pooled cotton root samples were obtained for each treatment. Frozen samples were ground to a fine powder in liquid nitrogen using a pestle and mortar. Total RNA was isolated with Plant RNA Kit (Tiangen, Beijing, China), and cDNA was synthesized with the RevertAid First Strand cDNA Synthesis Kit (Thermo Scientific, USA) following the manufacturer's procedure. The qRT-PCR was performed to quantify the transcript levels of the following genes: three key genes in the lignin metabolism pathway (peroxidase, *POD*; phenylalanine ammonia lyase, *PAL*; and polyphenol oxidase, *PPO*) and four pathogenesis-related (PR) genes (4-coumarate: CoA ligase, *4CL*; *basic chitinase*; *acidic chitinase*; and *β-1*,*3-glucanase*) with specific primers (Table 1); the cotton *ubiquitin* gene was used for normalization. Cycle thresholds were determined in three biological replicates per sample using LightCycler 480 system (Roche Diagnostics, Mannheim, Germany) and the SYBR Green Premixus Ex Taq TMII (Takara, Beijing, China) as the reporter dye. Amplification conditions consisted of denaturation for 10 min at 95°C followed by 40 cycles of 30 s at 95°C, 30 s at 60°C, and 30 s at 72°C. A final extension step was performed for 10 min at 72°C, followed by a melting curve program at 60°C to 95°C with an increase step of 0.5°C. Gene expression data were normalized using *ubiquitin*.

### 2.8. Statistical Analysis

Comparisons of treatment means for each experiment were conducted based on analysis of variance (ANOVA) and the SPSS 20.0 software; means were separated by Tukey's honestly significant difference (Tukey's HSD) test.

## 3. Results

### 3.1. In Vitro Antifungal Activity of the Endophytic Fungus CEF559 against *V. dahliae*

**Figure 1:**
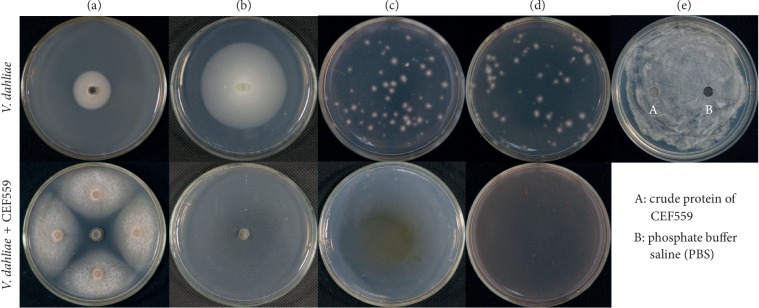
Growth inhibition of *Verticillium dahliae* by *Fusarium solani* CEF559: antifungal activity of *F. solani* CEF559 against *V. dahliae* in the dual culture test after incubation for 6 days (a); morphology of *V. dahliae* Vd080 incubated alone (top) and dual cultured with CEF559 on PDA media (bottom) for 14 days (b); inhibition of *V*. *dahliae* germination in the unsterilized (c) and sterilized (d) metabolites of CEF559; antifungal activity of crude protein *F. solani* CEF559 towards *V. dahliae* after incubation for 3 days (e).

The dual culture tests showed that CEF559 inhibited mycelial growth of *V*. *dahliae* (Figure 1(a)), with an average inhibition of 75%. Microsclerotia were produced on the edge of the dual culture colony, but not in *V. dahliae* only (Figure 1(a)). Nonvolatile metabolites of CEF559 also completely inhibited mycelial growth of *V*. *dahliae* (Figure 1(b)). However, *F. solani* CEF559 volatile compounds did not inhibit *V*. *dahliae* mycelial development (data not shown). CEF559 metabolites completely inhibited conidial germination of *V. dahliae* irrespective of whether the metabolites were autoclaved or not (Figures 1(c) and 1(d)). The crude protein of CEF559 did not show any antifungal activity towards *V*. *dahliae* (Figure 1(e)).

### 3.2. Effects of *F. solani* CEF559 on *V. dahliae* Sporulation

**Figure 2:**
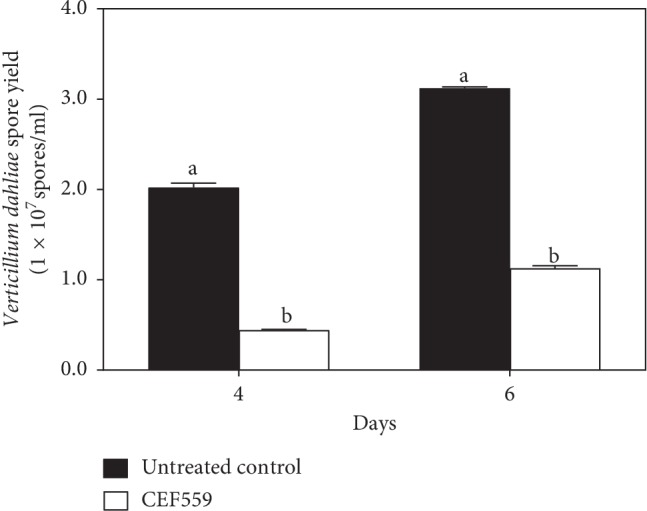
Effect of *Fusarium solani* CEF559 metabolites on sporulation of *Verticillium dahliae*. Means ± standard errors (*n* = 3) labeled with different letters indicate significant difference (*P* < 0.05) according to Tukey's honestly significant difference (Tukey's HSD) test.

Four days after *V*. *dahliae* Vd080 conidia were cultured with the cell-free culture filtrate of CEF559, the average conidial concentration was 4.4 × 10^6^ spores·mL^−1^, much less (*P* < 0.01) than the control 2.0 × 10^7^ spores·mL^−1^. On day 6, the corresponding values were 1.1 × 10^7^ and 3.1 × 10^7^ spores·mL^−1^ (Figure 2).

### 3.3. Suppression of Cotton Verticillium Wilt in Greenhouse

**Figure 3:**
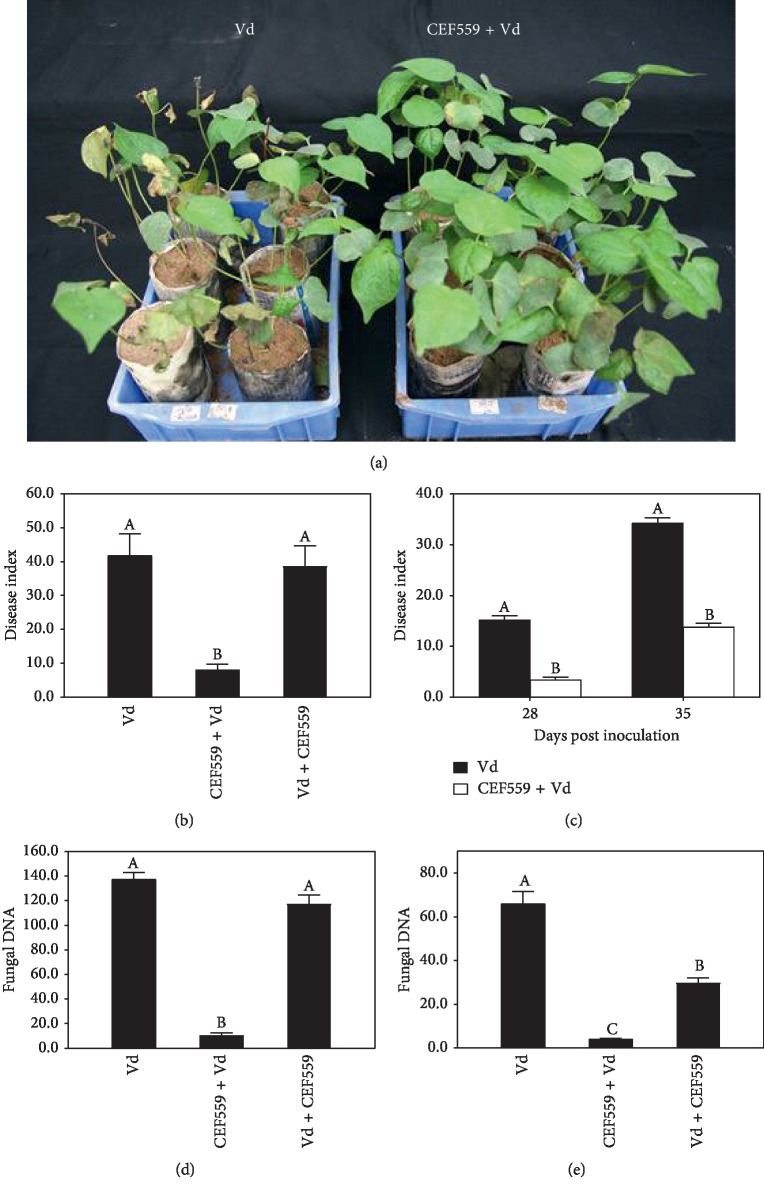
*Fusarium solani* CEF559 protects cotton plants from *Verticillium dahliae* infection. (a) Cotton plants of cv. Jimian11 were inoculated with *F. solani* CFE559 4 days before inoculation with *V. dahliae* strain Vd080 (Vd): photographed 21 dpi with *V*. *dahliae*. (b) Wilt index of cotton plants inoculated with *V. dahliae* Vd080 (Vd), inoculated with *F. solani* CFE559 liquid culture 4 days before inoculation with *V. dahliae* (CEF559 + Vd), and with *V. dahliae* 4 days before inoculation with *F. solani* CFE559 liquid culture (Vd + CEF559). (c) Wilt index of cotton plants inoculated with *F. solani* CFE559 solid culture 4 days before inoculation with *V. dahliae*. Quantified *Verticillium dahliae* DNA in roots (d) and hypocotyls (e) of cotton plants for three treatments as in (b). Data are means of three replicate experiments in greenhouse assay. Vertical bars represent standard errors of the means. Different letters indicate significant differences based on Tukey's HSD test (SPSS, v 20.0) at *P*=0.05.

In the liquid culture inoculation study, cotton plants treated with CEF559 4 days before inoculation with *V*. *dahliae* reduced (*P* < 0.05) wilt development when assessed 21 dpi (Figure 3(a)): disease index of 8.0 for the treated, compared to 38.8 for the control (Figure 3(b)). Inoculating with CFE559 after *V*. *dahliae* inoculation failed to reduce wilt development (Figure 3(b)). Similarly, in the solid culture inoculation, cotton plants inoculated with *F*. *solani* CFE559 4 days before inoculation of *V. dahliae* Vd080 reduced (*P* < 0.05) wilt development: the control efficacy was 77.6% and 59.9% when assessed 28 and 35 dpi of Vd080, respectively (Figure 3(c)). The qPCR results on *V*. *dahliae* biomass in the roots showed the same pattern as for the wilt index (Figures 3(b) and 3(d)). However, for *V*. *dahliae* biomass in the hypocotyls, CEF559 treatment after inoculation of *V*. *dahliae* also led to a significant reduction in *V*. *dahliae* biomass (Figure 3(e)).

### 3.4. Effect of *F. solani* CEF559 Inoculation on Cotton Seed Emergence and Seedling Development

*F*. *solani* CEF559 did not affect the growth of cotton plants (Table 2). The proportion of cotton seed emergence 5 days after sowing did not differ among treatments: 83.3% (CEF559) and 85.7% for the control. Similarly, no significant differences were observed between the two treatments in plant height, root length, and fresh weight when assessed 20 days after sowing, compared to the control (Table 2).

### 3.5. Field Suppression of Cotton Verticillium Wilt

**Figure 4:**
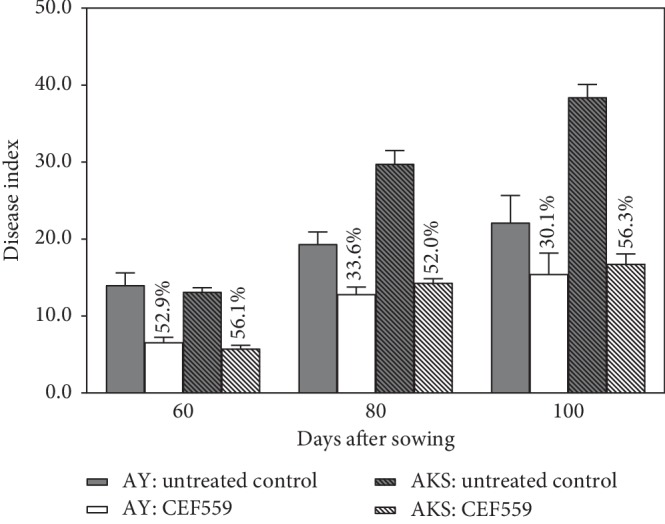
*Fusarium solani* CEF559 protects cotton plants from *Verticillium dahliae* infection at field sites in Anyang (AY) and Akesu (AKS) with a history of severe cotton wilt. Data are means of three replicate experiments in field assay. Figures above the bars are the average efficacy for the CEF559 treatment compared with the same site and date of investigation.

Wilt index increased over time at both sites (Figure 4). For the CEF559 treatment, the average disease index ranged from 6.6 to 15.5 and from 5.8 to 16.8 for Anyang and Akesu sites within 100 days after sowing, respectively, compared to the corresponding values for the control from 14.0 to 22.1 and from 13.1 to 38.4 (Figure 4). CEF559 treatment led to significant (*P* < 0.05) reductions in wilt index at both sites, with the control efficacy ranging from 30.1% to 56.3% (Figure 4).

### 3.6. Expression of Key Genes in the Lignin Metabolism Pathway and PR Genes

**Figure 5:**
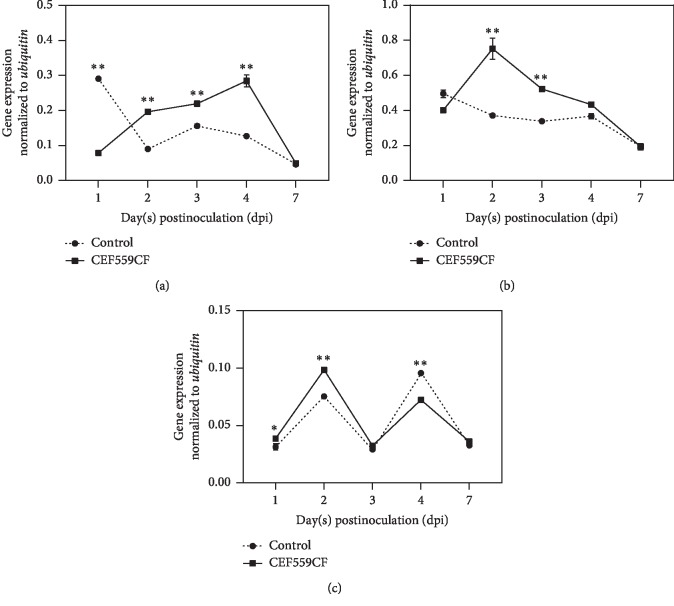
Effect of *Fusarium solani* CEF559 on the relative expression levels of three key genes in the lignin metabolism pathway in cotton roots after inoculation with *Verticillium dahliae*. Data are means of three replicate experiments in greenhouse assay. The bars represent the average induction (±SE) of gene transcripts normalized to the *ubiquitin* gene for three replicates. The vertical bars represent the standard error of the means. Comparisons of gene expression among treatments were conducted using a Tukey's HSD test (SPSS, v 20.0). Asterisks indicate statistically significant differences compared with control roots (double asterisks, *P* < 0.01; single asterisk, *P* < 0.05): (a) *PAL*; (b) *POD*; (c) *PPO*.

Although gene expression patterns varied greatly with time, compared with the untreated plants, the three key genes in the lignin metabolism pathway were all upregulated (*P* < 0.01) in the roots of those plants treated with CEF559 2 dpi compared with those plants inoculated with *V. dahliae* only (Figure 5). The expression of *POD* and *PPO* genes reached the peak 2 dpi, with the respective 2.0- and 1.3-fold increases over the control (Figures 5(b) and 5(c)). The expression of *PAL* reached the peak 4 dpi, ca. 2.2-fold increases over the control (Figure 5(a)). However, the gene expression level of *PAL* and *POD* genes 1 dpi and *PPO* 4 dpi showed a downregulation than the control (Figure 5).

**Figure 6:**
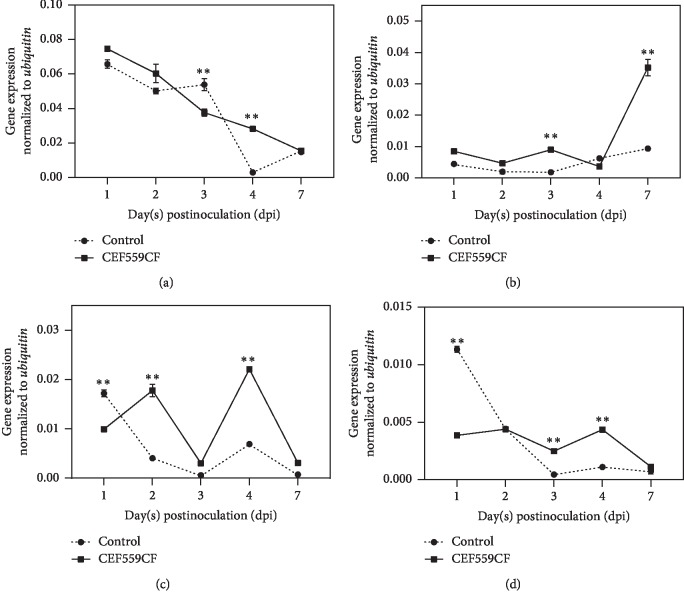
Effect of *Fusarium solani* CEF559 on relative expression levels of four pathogenesis-related (PR) genes in cotton roots after inoculation with *Verticillium dahliae* using quantitative reverse transcription PCR (qRT-PCR). Data are means of three replicate experiments in greenhouse assay. Vertical bars represent standard errors of means. Comparisons of gene expression among treatments were conducted using a Tukey's HSD test (SPSS, v 20.0). Asterisks indicate statistically significant differences compared with control roots (double asterisks, *P* < 0.01; single asterisk, *P* < 0.05): (a) *4CL*. (b) *Basic chitinase*. (c) *Acidic chitinase*. (d) *β-1,3-Glucanase*.

As for the key genes in the lignin metabolism pathway genes, CEF559 also induced PR gene expression in the treated roots. Except for *4CL*, the transcription level of *basic chitinase*, *acidic chitinase*, and *β-1,3-glucanase* were all upregulated on 3 dpi, compared with those plants inoculated with *V. dahliae* only, with increases of 4.9-fold, 5.0-fold, and 5.5-fold, respectively (Figure 6). The expression of *4CL* in treated plants was approximately 9.9-fold of the control 4 dpi (Figure 6(a)).

## 4. Discussion

Biological control of plant pathogens is a safer alternative to chemical treatments. In our previous study [24], 642 endophytic fungi were obtained from Verticillium wilt-resistant cotton varieties, and 80 of these endophytes were evaluated for their *in vitro* inhibition activity against *V*. *dahliae* isolate Vd080. Thirty-nine of these 80 isolates exhibited varying degrees of inhibition against *V*. *dahliae*. Of these 39 isolates, CEF818, CEF714, CEF642, and CEF193 were further tested for their biocontrol against cotton wilt in greenhouse and field trials [25]. In the present study, CEF559 strain, acquired from further screening for the strain pool excluding the tested 80 strains, can protect cotton plants against Verticillium wilt.

Over the last two decades, nonpathogenic *Fusarium* sp. isolates have been shown to have a varying degree of biocontrol potential against Verticillium wilt in several crop plants [12,16,27–29,36–39] although *F*. *oxysporum* and *F*. *solani* are also soilborne pathogens of some crop plants. For example, *F*. *oxysporum* strain F2 significantly reduced *V*. *dahliae* disease development in eggplants [16, 28, 29, 36]. *F*. *oxysporum* strain By125 and *F*. *oxysporum* strain CanR-46 achieved 69% and 92% control against cotton wilt in greenhouse, respectively [30, 37]. The present study suggested a new *Fusarium* strain (*F*. *solani* CEF559) could protect cotton plants against wilt with greater than 60% control in greenhouse studies, similar to the control level (50%) achieved by another *F*. *solani* strain (Bx215) in a greenhouse experiment [37]. Even in fields that were previously infested heavily with Verticillium wilt, this new isolate significantly reduced wilt development. For a given endophyte, biocontrol efficacy may depend on specific abiotic and biotic conditions [38]. Thus, it is not surprising that control efficacy in the present study varied between the two field sites.

Understanding the mechanisms involved in the antagonistic effects against plant pathogens is important for selecting effective and sustainable biocontrol strategies. In the present study, a dual culture of *V*. *dahliae* and *F*. *solani* CEF559 showed a decrease in the growth rate of the pathogen. Therefore, CEF559 could be acting at least partially through antagonistic interactions against *V. dahliae*. Similar results were also obtained from *F*. *oxysporum* strain F2, which has been reported to inhibit *V. dahliae* in eggplant by competition [29]; indeed, many nonpathogenic *Fusarium* strains can consume carbon sources more efficiently than pathogens [39]. Beneficial fungi can deprive pathogens of space and nutrients by colonizing the shared ecological niche [40]. CEF559 was isolated from cotton plants and, therefore, it probably can colonize cotton plants efficiently. Treating cotton roots with CEF559 reduced Verticillium wilt; in addition, the growth rate of CEF559 is greater than *V*. *dahliae*. Many biocontrol strains produce diverse biologically active secondary metabolites, which can inhibit pathogen mycelial growth and spore germination [41]. Various isolates of nonpathogenic *Fusarium* sp. have different competitive abilities against pathogens, and their indirect effects may vary. Nonvolatile metabolites of CEF559 also inhibited mycelial growth of *V*. *dahliae*, whilst volatile compounds did not inhibit *V*. *dahliae* mycelial development. Moreover, CEF559 metabolites completely inhibited conidial germination of *V. dahliae* irrespective of whether the metabolites were autoclaved or not. On the other hand, the crude protein produced by CEF559 did not show suppressive effect on the growth of *V. dahliae*. Therefore, we may conclude that the inhibitory activity of CEF559 is mainly due to the low molecular nonvolatile compounds instead of proteins and direct mycoparasitism.

Induced resistance is an important biocontrol mechanism [42]. Phenylpropanoid metabolic pathway in plants plays an important role in the plant defense response, and the enzymes of PAL, PPO, and POD were involved in the synthesis of lignin through this pathway [43]. Increased PPO, PAL, and POD activity was correlated with disease resistance in plants [44]. PPO and PAL activities are also important in plant disease resistance because they help to avoid oxidative damages [45]. In addition, the enhanced expression of defense-related enzyme genes contributes to the activation of defense system [46]. The level of enzyme activity may be related to the level of gene expression. For example, *Sporidiobolus pararoseus* strain Y16 treatment induced expression of these genes and increased enzyme activities, indicating a positive relationship of the gene expression level with enzyme activity [47]. Compared with those plants inoculated with *V. dahliae* only, *PAL*, *POD*, and *PPO* in root tissues were all significantly upregulated after CEF559 treatment, indicating that locally induced defense through the increased expression of these key genes in the lignin metabolism pathway is an important mechanism of CEF559 against *V*. *dahliae*. In addition, many plant resistance-related genes help to limit the growth and expansion of pathogens [48, 49]. In the present study, CEF559 induced a moderate increase of pathogenesis-related gene transcripts, including *basic chitinase*, *acidic chitinase*, *4CL*, and *β*-*1,3*-*glucanase*. Similarly, *F*. *oxysporum* isolate Fo47 can induce defense responses in several crops [26, 27]. *F*. *oxysporum* strain F2 induced the expression of defense-related genes *PR1* and *PR4* in eggplant, which was positively correlated with BCA population sizes in the rhizospheres [16]. Induced resistance in cotton by CEF559 in relation to innate resistance requires further research. Furthermore, additional research is needed to ascertain whether the host responses induced by CEF559 are conditional on the presence of *V. dahliae*.

The inherent biological complexity of the soil system has served as a challenge to the success of biocontrol of soilborne diseases. The rapid decline in the density of biological organisms typically encountered after introduction to the soil due to competitive inactions [50]. There has been an increasing interest in using the introduction of multiple BCAs to control soilborne disease with the component BCAs possessing different biocontrol mechanisms and ecological requirements [17, 18]. This could improve the survival of beneficial organisms in the temporally and spatially fluctuating rhizosphere environment and ensure that at least one of the beneficial microbes can survive. For example, a previous study has shown that the survival of introduced diverse probiotic consortia increased with increasing diversity, and high probiotic diversity could reduce pathogen density in the rhizosphere and decrease the disease incidence due to both intensified resource competition and interference with the pathogen [51]. Several cotton endophytic fungi isolates (including *F*. *solani* CEF559) showed biocontrol efficacy against cotton wilt [24, 25], and further research is needed to study their combined use to form a synthetic microbial consortium against cotton Verticillium wilt.

## Figures and Tables

**Table 1:** Oligonucleotide primers used in this study for reverse transcription quantitative PCR of defense genes and pathogenesis-related or reference genes from cotton.

Gene name	Primer sequence (5′ to 3′)
*β-1,3-Glucanase*	F: CACAGGTGCTGAAGTTGGT
	R: CGATGGAGGGAAAGATGA

*Basic chitinase*	F: CTTAGCCCAAACTTCCCA
	R: TACATTGAGTCCACCGAGAC

*Acidic chitinase*	F: GCTTTGATGGTTGTGCTCA
	R: CCACCCACCTGTAGTTTCA

*4CL*	F: ATTCAAAAGGGAGATGCC
	R: GAGAAGGGCAAAGCAACA

*PAL*	F: TGGTGGCTGAGTTTAGGAAA
	R: TGAGTGAGGCAATGTGTGA

*PPO*	F: ATATCCTTGTTCTGTCTGCTA
	R: CTCCTTCTACCGTCTCTTC

*POD*	F: CCGCATAACCATCACAAG
	R: ACTCTCATCACCTTCAACA

*Ubiquitin*	F: GAGTCTTCGGACACCATTG
	R: CTTGACCTTCTTCTTCTTGTGC

**Table 2:** Effect of *Fusarium solani* CEF559 on cotton emergence and biomass.

Treatment	Emergence rate (%)	Plant height (cm)	Root length (cm)	Fresh weight (g)
CK	85.67a	10.2 ± 1.26a	9.6 ± 0.65a	0.98 ± 0.11a
CEF559	83.33a	9.9 ± 0.652a	9.5 ± 0.791a	1.12 ± 0.114a

Data (mean ± SE) in the table are the averages of three biological experiments. Data followed by different letters indicate statistically significant differences at the level of *P* < 0.05 according to Tukey's HSD.

## Data Availability

The data used to support the findings of this study are available from the corresponding author upon request.
